# Fake News Affecting the Adherence of National Response Measures During the COVID-19 Lockdown Period: The Experience of Vietnam

**DOI:** 10.3389/fpubh.2020.589872

**Published:** 2020-09-23

**Authors:** Thao Thi Phuong Nguyen, Duy Cao Nguyen, Anh Trong Tung Nguyen, Long Hoang Nguyen, Giang Thu Vu, Cuong Tat Nguyen, Trang Ha Nguyen, Huong Thi Le

**Affiliations:** ^1^Institute for Preventive Medicine and Public Health, Hanoi Medical University, Hanoi, Vietnam; ^2^Institute of Health Economics and Technology, Hanoi, Vietnam; ^3^Center of Excellence in Evidence-Based Medicine, Nguyen Tat Thanh University, Ho Chi Minh City, Vietnam; ^4^Institute for Global Health Innovations, Duy Tan University, Da Nang, Vietnam; ^5^Faculty of Medicine, Duy Tan University, Da Nang, Vietnam

**Keywords:** fake news, COVID-19, lockdown, Vietnam, response measures

COVID-19 has severely affected people's health and well-being, including all economic sectors, tourism, culture, and education. Along with combating the COVID-19 epidemic, fighting the “infodemic,” which refers to the rapid spread of misinformation, related to the disease on the social media is also of concern, because fake news spreads faster and easier than this virus, and just as dangerous (1). This causes anxiety among public and motivate them to follow or believe in the unreliable information, making the containment of epidemic more complicated when raising the uncertainty and unnecessary behavior of public, and hindering the collaboration and unity in combating the epidemic (2).

In Vietnam, the role of press and social media in spreading the government's information regarding COVID-19 pandemic is undeniable; however, fake news phenomenon is still substantial. A report of the Ministry of Information and Communications revealed that from 01/02 to 05/31/2020, the press had published a total of 560,048 news and articles about COVID-19 translation. According to the statistics of the police force, from the onset of the Covid-19 epidemic to the middle of March 2020, there were nearly 300,000 news articles on cyberspace, posts on websites, blogs, forums, and almost 600,000 news, articles, videos and clips related to the disease posted on social networks (3). When Vietnam entered a “new normal” state after April 17, the rate of news articles related to the COVID-19 epidemic was still maintained by media and radio agencies around 28–40% of news and articles on recovery, economic development but not subjective in disease prevention (3). In the first 5 months of 2020, there have been nearly 17 million mentions in the Vietnamese cyberspace (status lines, comments) related to the COVID-19 epidemic situation in Vietnam (3). However, all of these swift measures were not enough to regain the public's trust and stop the rapid spreading of fake news through the population. There were various news, articles with unverified, distorted, false content that attracted millions of comments and shares. Security authorities have verified and treated 654 cases of reporting fake news, sanctioned administratively more than 146 people (4). Cyberspace is a favorable environment for fake news given that 64 million Vietnamese are Internet users, as well as 58 million people have at least one social network site account (e.g., Facebook, Instagram, or Zalo) (5).

Massive media bombardment regarding the lockdown period led to public speculation in Vietnam, as in many parts of the world, which was the cause of grocery shortages and great consequences. Supermarkets and grocery stores revealed out-of-stock of antibacterial gels, antibacterial wipes, detergents, and toilet paper, while pharmacy stores reported the shortage of isopropyl alcohol, latex gloves, and medical-grade masks (6), leading to the deficiency of personal protective equipment in hospital settings, including sites designated by the MoH as COVID-19 response sites (7). Increasing fake news regarding alternative COVID-19 treatments has led people to storm pharmacies and buy stocks of available drugs such as hydroxychloroquine (8); consequently, many patients with systemic lupus erythematosus (SLE) and rheumatoid arthritis (RA) cannot access their treatment because of nationwide shortages. Given that self-medication is common among the Vietnamese due to the lack of governmental regulation about drug use, these drugs are uncontrolledly purchased and used without a prescription, which increase the risk of hospitalization due to drug misuse (9). Moreover, spreading fake information on the number of confirmed cases and fatal cases in Vietnam led to the public's anxiety and stress. Some false information about border closure with China, calling on Vietnam to close the border, calling for people to go on strikes throughout the territory of Vietnam, or disseminating misinformation about the vaccine against Coronavirus affects significantly destabilizing security and politics in Vietnam (5). Fake news affected sharply to stigma among unskilled labor groups and their family in ethnic minority groups. Lack of COVID 19 epidemic knowledge and fear of stigma (include their family was attacked and alienated by false information) that commenced to evading health declaration procedure. In consequence, increasing the number of unconfirmed COVID-19 cases will become dangerous spreading sources for their ethnic minority groups in particular and all community in general (10).

In response to fake news, especially during the COVID-19 pandemic, the government has made early predictions and concrete strategies. Since June 12, 2018, the National Assembly of Vietnam passed a cybersecurity law comprising seven chapters and 43 articles that stipulated activities to protect national security, ensure social order and safety on cyberspace, and responsibilities of agencies, organizations, and individuals involved (11). Cybersecurity law made it easier for the government to handle violations of organizations and individuals on cyber such as posting and spreading fake news. As a result, over the past time, according to the Vietnamese Ministry of Public Security statistics, the police force has made a list of hundreds of objects, convened, fought nearly 200 cases, and administratively handled more than 30 cases of spreading fake information of COVID-19 epidemic (3). Secondly, the government has now established official communication channels on social networking sites such as the Government Information page on Facebook or the official page of the Ministry of Health on Zalo—one of the most popular social applications in Vietnam. Besides, ministries and departments have directly sent messages to people's contact phone numbers to provide information about the epidemic, which was never implemented before. This solution helps all Vietnamese people who do not have the opportunity to access the internet to capture information in time about the pandemic, thereby distinguishing fake and accurate news. In ethnic minority groups, local authorities enhanced advocacy on prevention measures, increasing knowledge of the COVID 19 epidemic, avoiding stigma COVID 19 patients and their family (10). Finally, accompanying the people in preventing and eliminating false information on cyberspace, the investigating police agency has intensified the review, discovered, and promptly sanctioned cases of giving incorrect information for profiteering purposes or confusing public opinion.

Moreover, the government needs to pledge to be transparent in providing information, helping people grasp promptly and take measures to prevent and fight epidemics. Solving this method also helps people have faith in the official news of the state. However, the most important thing is that every internet user needs to be alerted to select reliable information and respect seriously for Vietnamese law. Furthermore, health professionals and health workers should regularly transfer necessary knowledge about disease prevention and control to people on the social network.

## Author Contributions

TTPN, DCN, ATTN, and LHN: conceptualization. TTPN, DCN, and ATTN: writing—original draft. LHN, TTPN, DCN, ATTN, GTV, CTN, THN, and HTL: writing—review and editing. CTN, THN, LHN, and GTV: project administration. All authors contributed to the article and approved the submitted version.

## Conflict of Interest

The authors declare that the research was conducted in the absence of any commercial or financial relationships that could be construed as a potential conflict of interest.
